# Supplementary material for: Advances in Management and Therapeutics of Cutaneous Basal Cell Carcinoma
Source: Cancers (Basel). 2022 Jul 30;14(15):3720. doi: 10.3390/cancers14153720 (PMC9367462; doi:10.3390/cancers14153720)
Supplement: Supplementary file 1 [file cancers-14-03720-s001.zip › cancers-1820863-supplementary.pdf]

Supplementary Table S1. Risk-stratified clinicopathologic features of basal cell carcinoma.

	Lower-risk BCCs	Higher-risk BCCs
**Histologic features**	▪ Growth pattern: Superficial, Nodular<br>▪ Absent perineural invasion	▪ Growth pattern: morpheaform, infiltrative, micronodular, sclerosing, basosquamous<br>▪ Present perineural invasion<br>▪ Gross bone invasion
**Anatomic locations**	▪ Trunk and extremities, excluding pretibial surface, hands, feet, nail units, and ankles	▪ Medium risk: cheeks, scalp, forehead, neck, jawline, pretibial surface<br>▪ Higher risk: face (other areas), ears, hair-bearing lip, postauricular skin, hands, feet, nipples/areola, genitalia, nail units
**Size in greatest dimension**	▪ Low risk: $\leq$ 2 cm	▪ Medium risk: >2 cm and $\leq$ 4 cm<br>▪ Higher risk: > 4 cm
**Patient comorbidities**	▪ No immunosuppressive conditions<br>▪ No cancer-predisposing genetic syndromes	▪ Immunosuppressive conditions<br>▪ Basal cell nevus syndrome, xeroderma pigmentosum, and other syndromes at high-risk for skin cancer
**Occurrence**	▪ Primary	▪ Recurrent
**Invasiveness**	▪ Less invasive: localized tumors	▪ Invasive: Locally-advanced BCC, metastatic BCC
